# EQ-5D-5L in the General German Population: Comparison and Evaluation of Three Yearly Cross-Section Surveys

**DOI:** 10.3390/ijerph13030343

**Published:** 2016-03-21

**Authors:** Manuel B. Huber, Peter Reitmeir, Martin Vogelmann, Reiner Leidl

**Affiliations:** 1 German Research Center for Environmental Health, Institute for Health Economics and Health Care Management, Helmholtz Zentrum München, Postfach 1129, Neuherberg 85758, Germany; reitmeir@helmholtz-muenchen.de (P.R.); leidl@bwl.lmu.de (R.L.); 2 Wort & Bild Verlag Konradshöhe GmbH & Co. KG, Baierbrunn 82065, Germany; martin.vogelmann@wortundbildverlag.de; 3 Munich Center of Health Sciences, Ludwig-Maximilians-University, Ludwigstr. 28 RG, Munich 80539, Germany

**Keywords:** EQ-5D-5L, cross-section, reference values, Germany

## Abstract

Health-related quality of life (HRQoL) is a key measure for evaluating health status in populations. Using the recent EQ-5D-5L for measurement, this study analyzed quality of life results and their stability over consecutive population surveys. Three cross-section surveys for representative samples of the general German population from 2012, 2013, and 2014 were evaluated using the EQ-5D-5L descriptive system and valuation by the Visual Analog Scale (VAS). Aggregated sample size reached 6074. The dimension with the highest prevalence of problems was pain/discomfort (31.7%). Compared with 2012 (59.3%), the percentage of participants in the best health state increased slightly in 2013 (63.4%) and 2014 (62%). Over the 3-year period, diabetes and heart disease had the strongest negative influence on mean VAS result. The number of reported chronic diseases cumulatively reduced mean VAS. Extreme problems in one or more dimensions were stated by only 0.1%–0.2% of patients. Of the potential 247 health states with a problem score ≥20, only six were observed in the aggregated sample. HRQoL results were fairly stable over the 3 years, but the share of the population with no problems was not. Results from the aggregated sample may serve as updated reference values for the general German population.

## 1. Introduction

Health-related quality of life (HRQoL) is one key measure for the effectiveness and cost-effectiveness of healthcare interventions. HRQoL can be measured by different instruments including the EuroQol five-dimension questionnaire (EQ-5D) [1]. The EQ-5D is a well-accepted and widely used measure. Its use, quantified by PubMed search results, has steadily increased on a year by year basis since 1992 [2]. The EQ-5D is one of the psychometric approaches and is a descriptive generic instrument. It consists of five dimensions (mobility, self-care, usual activity, pain/discomfort, anxiety/depression) and a visual analog scale (VAS). The VAS is a continuous response scale that ranges from 0 (worst state) to 100 (best state). Responders rate their current health state by selecting a point on this scale. The 3L version of the EQ-5D includes three answer levels for each of the five dimensions, ranging from no problems (1) to some problems (2) to extreme problems (3). The newer 5L version incorporates five answer levels (1–5), with additional slight as well as severe problem categories, and was introduced in 2011 [3]. The best health state is depicted by 11111 and the worst by 55555. The advantages of the EQ-5D-5L compared with 3L include reduced ceiling effects [4,5], increased level of detail [6], and more discriminatory power while establishing convergent groups and their respective validity [7,8]. The responses for each dimension deliver a profile, which is then valued by VAS, but VAS results are also usually reported when no valuation is done. Alternatively, a population-based value set may be used. Examples of derived and well-accepted value sets that generate utilities by time trade-off valuation of given health states for the 3L version include those by Dolan 1997 [9] for the UK or Greiner *et al.* 2005 [10] for Germany; other value set concepts are based on experienced health such as those for Sweden [11] and Germany [12]; value sets for the 5L version are under development [13] and currently only available for England, Japan, Canada, and Uruguay [14]. This study only considers the VAS valuation of their own health state by respondents, thus establishing their perspective on quality of life.

Longitudinal data are harder to obtain but also have certain disadvantages over cross-sectional data, including correlation of observations among one individual. Thus, they require sophisticated handling [15]. Typically [16], general population surveys using the EQ-5D are conducted as single cross-sectional surveys and are eventually repeated after several years. Using single-year samples usually assumes stability of the results found and, for many survey parameters, sample sizes ensure this assumption in well-designed studies. Empirically, however, temporal stability of the results of such cross-sectional studies has hardly been investigated. Focusing on this research gap, the aim of the study is to analyze the results of three EQ-5D-5L surveys conducted in 2012, 2013, and 2014 by focusing especially on variation in results of health state descriptions and valuations, including the role of sociodemographic parameters and one important determinant of HRQoL, chronic diseases [17,18]. Results are compared with previous surveys using the 3L version, and may lead to updated reference values of the 5L version for the general German population.

## 2. Methods

### 2.1. Data Sampling

Quality of life measured by the EQ-5D is an add-on component to a yearly survey of the general German population that is conducted by the IFAK research institute on behalf of the Wort & Bild Verlag. Results from earlier add-on studies using the EQ-5D-3L have been published elsewhere [12,18,19]. The Wort & Bild survey uses a random-route procedure to randomly select a sample of the German population aged 14 years and older. The sampling system contained over 53,000 sample points with a minimum of 350 households per point. On average, the sample points included 700 households. The sample points were layered according to region and the Germany specific BIK code, a geographic and demographic classification system. To achieve representability 516 sample points were randomly chosen from these layers. The interviewers were equipped with a starting address in these sample points and had to follow randomly chosen routing information. They were instructed to ask for participation at every fifth household on this route, until five surveys were completed. Household members were selected based on a Kish selection grid, and were asked for consent to take part in the survey. Response rates were high at 72.6% in 2012, 71.8% in 2013, and 70.3% in 2014. The main reason for non-responsiveness was the absence of household members. Over 2000 surveys were completed each year. The survey data, collected in 2012, 2013, and 2014, were quite representative of the German population (see supplementary Table S1). Thus, weights were not applied for the purpose of this study. Sociodemographic parameters, health status, and distribution-based data were also extracted for this add-on study. Disease affliction is based on open-ended self-report; participants stated which diseases they suffered from during the last 3 months and whether these were chronic. Only the five most prevalent diseases in the total sample were evaluated for the purpose of this study.

### 2.2. Data Analysis

Descriptive methods were used to analyze the majority of data. Chi-square tests were used to evaluate frequency differences between survey years. Analysis of variance (ANOVA) was used to determine estimators for VAS reduction stratified by disease. All analyses, including problem score evaluation, are based on the statistical software package SAS 9.3 (SAS Institute Inc., Cary, NC, USA). Figures were created by using the package ggplot2 [20], version 2.0.0 (Hadley Wickham), for the software environment R [21], version 3.2.2 (R Foundation for Statistical Computing, Vienna, Austria).

## 3. Results

### 3.1. Study Sample

Table 1 shows the basic characteristics of the three evaluated samples. EQ-5D-5L data were available for 6074 people (2012: 2045; 2013: 2028; 2014: 2001), who were included in the analysis. In total, 52.9% of the participants were female. The average age was 47.1 years. The participants from 2013 (45.9 years mean age) were about 1.5 years younger than the participants from 2012 and 2013, which is indicated by fewer people in the age groups from 60 years onward. The number of people living alone stayed nearly the same, and a slight shift toward better education could be observed. The number of people with low education (high school with and without apprenticeship) decreased, whereas the number of people with medium (middle school) and high education (grammar school with and without university attendance) increased slightly. Missing education or living status data did not exclude participants from further analysis regarding HRQoL.

### 3.2. EQ-5D Distribution

EQ-5D-5L-related answer frequencies based on dimension are depicted in Table 2. The dimension with the least reported problems was self-care, followed by usual activity and anxiety/depression as well as mobility. In contrast, a high proportion or around one third of participants experienced problems with pain/discomfort. The chi-square test for statistical differences among the 3 years yielded a significant *p*-value (*p* = 0.04) only for pain/discomfort. The participants from 2013 and 2014 suffered from significantly fewer problems in this dimension.

### 3.3. Problem Score Data

The problem score (Table 3) aggregates the manifestations of each dimension by adding up the five answer levels into one number. In total, 3125 health states are possible for the EQ-5D-5L, but only 265 different health states were observed in our samples. An increasing problem score correlated with a decreasing number of actual observations. The problem scores ≤12 cover around 97% of participants, whereas the number of possible health states increases up to problem score 15. The number of realized different health states peaked at problem score 10. Calculating the overall *p*-value (chi-square test) for the influence of year on problem score delivered no significant result (*p* = 0.2165).

### 3.4. Most Prevalent Health States

**Figure 1:**
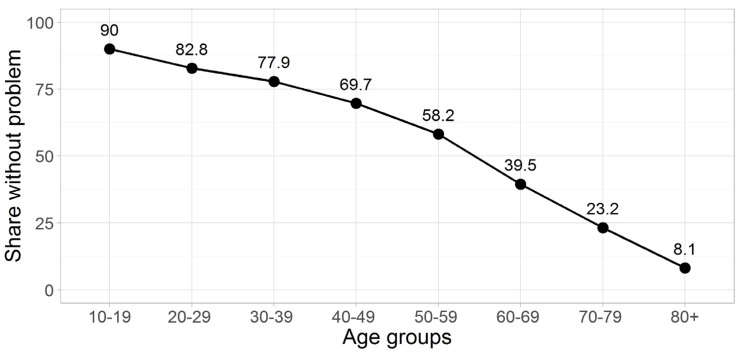
Percentage of respective age group without problem (EQ-5D-5L descriptive score: 11111), aggregated sample.

A majority of 61.6% stated that they had no problems (11111) in all five dimensions of the EQ-5D (Table 4). This manifestation was strongest—probably because of mean age difference—in 2013 at 63.4% and lowest in 2012 at 59.3%. The 11111 state is followed by 8.3% of participants reporting slight problems in the pain/discomfort dimension (11121) and 4.1% reporting slight problems in the anxiety/depression dimension (11112). The 11121 state dropped 0.7 percentage points from 2012 to 2014. At the same time, the 11112 state increased 1.3 percentage points from 2012 to 2014. However, the only significant differences between the 3 years were seen for the optimal health state and 11122 (slight problems with pain/discomfort and anxiety/depression). Figure 1 illustrates the percental decline over age groups in the share of respondents not reporting any problem. While around 90% of adolescents state to be in the optimal health state, only around 8% do so in the age group 80+.

### 3.5. VAS

**Figure 2:**
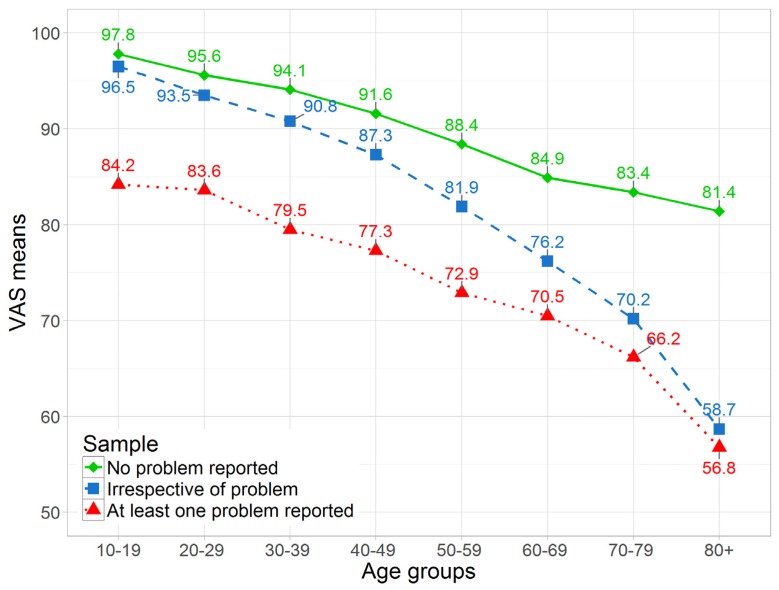
VAS means based on age group, stratified by optimal and non-optimal health state, aggregated sample. No problem reported shows the average VAS score for the subsample of participants with a descriptive score of 11111 (*n* = 3739). At least one problem reported refers to the subsample of remaining participants with at least one dimension score >1 (*n* = 2335). Irrespective of problem depicts the whole sample (*n* = 6074).

Figure 2 illustrates VAS means stratified by whether or not any problem was reported in the five descriptive dimensions of the EQ-5D. “No problem” refers to all 3739 participants with a score of 11111 and scores >11111 are referred to as “at least one problem reported”. Participants with no problem had a mean VAS score of 91.86. “Irrespective of problem” depicts the whole sample of 6074 participants. The VAS mean for all participants was 84.3 (women: 83.1; men: 85.7). It should be noted that EQ-5D scores do not imply the presence or absence of self-reported disease; they only measure the perception of health on the day of the survey. The reported VAS values become smaller with increasing age. Significant divergence is present for people with problems and those without. From age groups 10–19 to 70–79 years, the difference between both groups amounts to around 20 points. From age group 70–79 to 80+ years, a big decrease of around 10 points is observed for the problem group, whereas the group without problems deteriorates only by around 2.5 points. This resembles a rapid and significant worsening of the average respondent’s perception of their health status after age 75. The number of people with no problems (*n* = 3739) decreases continuously. In the age group 80+ years, fewer than 10% report no problems. Compared with men, women tend to have slightly lower VAS means from age groups 20–75 years and slightly higher means from around 75 years onward (not shown). The differences in the decrease in HRQoL between all three surveys and respective age groups are significant (*p* = 0.0072). Detailed results are available from the authors.

VAS values by age and sex group for the aggregated sample are reported in supplementary Table S2 and may be used as reference values.

### 3.6. Disease Affliction and Influence on HRQoL

Besides reporting problems in the EQ-5D dimensions participants were also asked to state their current chronic diseases. Compared with 2012 and 2014, participants from 2013 reported the lowest percentage of disease affliction, except for rheumatism at 1.6% in 2013 and 2014 (Table 5). This is likely to be rooted in the fact that the average age in the sample from 2013 was lower than in the other two samples. Overall, 4.4% stated that they suffered from musculoskeletal disease, 6.1% from hypertension, 3.2% from diabetes, 1.9% from heart disease, and 1.5% from rheumatism. From the total sample, only a minority of around 14% stated to suffer from at least one of these diseases.

Chi-square tests were used to determine statistically significant differences for the evaluated parameters in Table 5 across survey years, but none were found.

**Figure 3:**
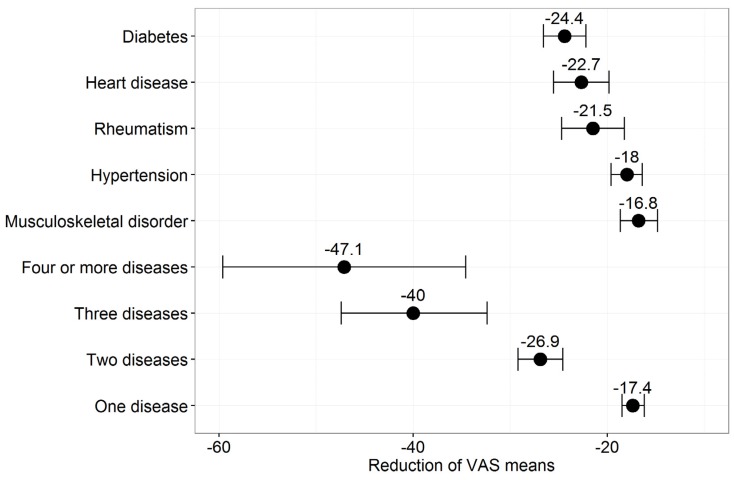
Reduction in VAS means by presence of disease, aggregated sample, disease as binary (yes/no) variable, disease evaluated in quantitative and qualitative form. Number of diseases refers to the five most prevalent diseases. Confidence intervals are shown alongside markers.

Thus, we evaluated the influence of diseases on HRQoL by aggregated sample. Figure 3 depicts the average reduction in VAS values (based on ANOVA) when disease is present. From a quantitative perspective, the presence of two diseases has a stronger negative influence on VAS mean than any of the single diseases. Due to the low number of observations for three and four diseases, the confidence intervals are very wide. Group affiliation (yes/no) was the only classification variable in this model. The widths of the 95% confidence intervals corresponded to the prevalence of the observed parameter. The higher the prevalence, the smaller the confidence interval. The VAS mean for having no chronic disease was 87.1 compared with 84.3 for the whole sample. Diabetes had the strongest negative influence on mean VAS value and led to a mean reduction of around 24.4 points. Of the five evaluated diseases, musculoskeletal disease had the lowest delta of around −16.8. The differences for the reduction in VAS means by disease, between years, were not significant (not shown, detailed results available from the authors). The reductions seem clinically relevant as they all exceed the minimal important differences that were identified for the EQ-5D in patients with back pain, early rheumatoid arthritis, or myocardial infarction [22].

## 4. Discussion

We found that the percentage of people in the optimal health state increased from 59.3% in 2012 to 63.4% and 62% in 2013 and 2014 respectively. This variation over all 3 years was significant (*p* < 0.02), but may be linked to the variation in sociodemographic data across the samples. To confirm this possible trend toward better health, more EQ-5D-5L data and especially follow-up evaluations are needed in future. Our results indicate that the survey approach, including the random-route selection process, is not suitable to deliver sufficient data for participants with extreme problems. It became apparent that sample sizes of 2000 participants, if selected by random-route procedures as in this study, are sufficient to analyze EQ-5D-5L data for problem scores ranging from 1 to 10 or even 15 but lack sufficient observations for higher scores. Only 0.1%–0.2% of participants used answer level 5. Severely ill people may not be able to participate in the questionnaire, may decline to participate, or may be absent from home. Research is needed to evaluate EQ-5D-5L in clinical patient populations with severe and extreme problems. Of the potential 247 health states with a problem score ≥20 in the 5L, only six were observed in this total sample of 6074 people. In order to increase inclusion of participants with extreme health problems, extension or supplements to the current sampling procedure may have to be considered. It becomes clear that the increased discriminatory power of the EQ-5D-5L may, at the same time, have drawbacks including lower numbers of extreme health states being observed. This problem increases in relevance when samples from the general population are used and selection bias toward the omission of severely ill participants is not accounted for.

Slight problems with pain/discomfort were reported less often, whereas slight problems with anxiety/depression increased over the 3-year period. However, the differences among all 3 years were not significant. In line with previous and other studies [18,23], pain/discomfort is the dimension with most problems.

Despite an optimal health state, VAS values deteriorated continuously from 97.8 in the age group 10–19 years to 81.4 in the age group ≥80 years. One explanation for this discrepancy are differences in HRQoL coverage of VAS and the five dimensions of the EQ-5D. The daily activities of an 80 year old, to a very high probability, are not the same as the daily activities of someone in his/her 40 s. These participants likely adapted their activities to their bodily constitutions. Therefore, the 80 year old’s ability to fulfill all current daily activities may still constitute “no problems”, while his/her overall fitness has actually depreciated over time. Owing to its dimension-free character, the VAS can pick up dimension-unspecific elements of HRQoL status.

Overall variation in results for health state descriptions and valuations, including sociodemographic parameters and chronic diseases, across the three surveys was low. Concerning underlying socioeconomic variables, we observed a trend toward a higher level of medium and high education (*p* < 0.002), but it could not be determined to what degree, or if at all, this was caused by lowered entry barriers and reduced standards for medium and high education.

### 4.1. Morbidity

Our results for the prevalence of diseases—qualitative and quantitative—were statistically not significantly different among the three surveys. Of the five diseases under evaluation, hypertension had the highest prevalence with 6.1%. This is in sharp contrast to Balijepalli *et al.* [24], who stated that 54.8% of adult primary care attendees suffer from high blood pressure. The primary care setting may be too restrictive. From a nationwide perspective Neuhauser *et al.* [25] conclude that between 2008 and 2011 around one third of the adult German population (age 18 to 79) had high blood pressure. This discrepancy may partly be rooted in age differences as our study included respondents from 14 years on, and, compared with the German population, has relatively low shares of respondents aged 80 years and older who are likely to suffer from some chronic disease. Furthermore, it is known that the prevalence of hypertension is underestimated by self-report for the disease [26]. Our results for the prevalence of Rheumatism and Diabetes differ, too. 1.6% of our participants claim to have rheumatism, while the actual prevalence seems to be around 2.5% [27]. The actual prevalence of diabetes is around 7% [28], while our surveys include 3.2% of participants who claim to suffer from diabetes. Thus, we would highly recommend clinical testing for evaluating respective disease prevalence. More importantly, we found out that the number of diseases cumulatively reduce VAS means. The presence of two diseases has a stronger influence on VAS mean reduction than any of the most prevalent diseases under evaluation (e.g., diabetes −24.4 *vs.* presence of two diseases −26.9). The reduction of VAS means increases rapidly to −40 for three diseases but the confidence intervals widen due to decreasing number of observations. Compared to Heyworth *et al*. [29] (UK) the VAS-means stratified by number of diseases mirror each other well (69.4, 63.3, 56.7, 40.7 *vs*. 66.9, 57.4, 44.3, 37.2 for 1, 2, 3 and 4 diseases respectively). Age and disease differences (asthma, COPD, Diabetes, Hypertension, ischemic heart disease and stroke) may partly explain the remaining divergences. Considering evidence of both studies it can be concluded that multimorbidity leads to patients with special medical needs.

### 4.2. Comparison with Other Studies

A number of baseline variables (aggregated sample) in this study can also be compared with earlier studies using the EQ-5D-3L in yearly population surveys by Wort & Bild Verlag from 2006 to 2011 (*n* = 11,177) [18]. The gender distribution in these surveys was comparable to our study. Low education decreased from 44.0% (2006–2011) to 39.7% (2012–2014), while medium and high education increased from 56.0% to 60.4% respectively. Fewer people lived with a partner (68.42% *vs.* 58.6%). The prevalence of disease fell among all categories. The strongest reduction was seen for musculoskeletal disease, which dropped from around 7.2% to 4.4% in our evaluation. Presence of chronic disease fell from 24.5% to 21.6%.

Comparable data for the EQ-5D-3L from 2006 and 2007 showed that the number of observed health states was 49, whereas the maximum number of possible health states was 243 [12]. This resembles a ratio from observed to possible states of around 1:5. The same ratio increased to around 1:12 in our study.

In a representative sample of the general German population of 2011 (*n* = 2469), Hinz *et al.* [30] identified 210 distinct health states in the 5L version, about a fifth less than the number of states identified in this study in a sample more than twice as large. Hinz *et al.* also reported that 47.5% (mean age 50.5 years) of the sample were in the optimal health state, which is lower than in this study but can be partly attributed to the higher mean age. The distribution of VAS values in the same age categories was quite stable, and the decline in VAS values by increasing age [31] resembles the proportions from 2006 [19]. Age-related deterioration in VAS values and health status is seen in other studies [32,33,34] for men and women as well as people with problems and those without. Our participants in the best health state showed an average VAS score of 91.9. This score is around 4 points higher than the 88 points in the survey from 2006 [19]. From age group 70–79 to age group 80+ years, a significant decrease in VAS means can be observed for those people who reported problems. Compared with Koenig *et al.* [35], who did not differentiate between genders, our current VAS mean of 92.2–95.1 for the age group 20–29 years (*n* = 842) is significantly higher than their VAS mean of 85.4 for the age group 18–24 years (*n* = 263). Mielck *et al.* [19] also reported lower VAS means for the youngest age group as well as overall (80.0 *vs.* 84.3). VAS means seem to drop to around or below 60 for the highest age group in all three studies. Mielck *et al.* [19] stated that 60.6% of participants were in the optimal health state (11111), which is slightly less than in our sample (61.6%).

It was difficult to find other consecutive, representative cross-sectional studies for Germany that used the EQ-5D-5L. A recent study by Steiber [36], incorporating the SF-12, reported that physical fitness decreased and fluid intelligence increased in a representative sample of older people from 2006 to 2012. Although fluid intelligence was not measured in our study, we saw an increase in people with no problems among all dimensions. A small fall was observed for people with no mobility problems from 27.4% (2012) and 27.9% (2013) to 27% (2014).

Limitations of this study include possible selection bias found in general population surveys. Clinical populations with severe or extreme problems were not observed. Sufficient data for severe or extreme problems are generally hard to come by using the current selection process. Furthermore, self-reporting of disease included no form of verification. True disease affliction may differ from self-reported answers. Yet, for the part of the general population that can be addressed by surveys, this study delivered useful norm data for three consecutive years. Respective evaluations are rare but are needed to determine the health status of the general German population.

## 5. Conclusions

Contrary to assumed stability, a small but significant trend toward better health was observed in three yearly surveys from 2012 to 2014. Remaining variation in results for health state descriptions and valuations, including sociodemographic parameters and chronic diseases, was low. The number of present diseases reduced VAS results cumulatively. The participant selection process, based on a random-route procedure, failed to deliver sufficient data for participants with severe or extreme problems. Given the extended size of the pooled sample, health state valuations identified here may serve as broadly founded reference values for the general German population.

## Figures and Tables

**Table 1:** Study population, education, and living status.

Parameters	2012	2013	2014	All
Mean age (years)	47.6	45.9	47.4	47.0
Male	977 (47.8)	950 (46.8)	932 (46.6)	2859 (47.1)
Female	1068 (52.2)	1078 (53.2)	1069 (53.4)	3215 (52.9)
Education *				
Low	854 (43.2)	733 (37.7)	730 (38.0)	2317 (39.7)
Medium	716 (36.2)	806 (41.5)	777 (40.5)	2299 (39.4)
High	409 (20.7)	404 (20.8)	412 (21.5)	1225 (21.0)
Living alone				
No	1159 (58.6)	1126 (58.0)	1135 (59.1)	3420 (58.6)
Yes	820 (41.4)	817 (42.0)	784 (40.9)	2421 (41.4)

Numbers as absolute values. Percentage in parentheses, if appropriate. * *p* < 0.05, chi-square test for statistically significant differences over the 3-year period.

**Table 2:** Answer frequencies based on dimension, aggregated sample.

Level	Mobility	Self-Care	Usual Activity	Pain/Discomfort	Anxiety/Depression
**1**	4998 (82.3)	5712 (94.0)	5274 (86.8)	4149 (68.3)	4987 (82.1)
**2**	656 (10.8)	260 (4.3)	567 (9.3)	1301 (21.4)	799 (13.2)
**3**	304 (5.0)	79 (1.3)	180 (3.0)	521 (8.6)	236 (3.9)
**4**	107 (1.8)	19 (0.3)	46 (0.8)	91 (1.5)	45 (0.7)
**5**	9 (0.1)	4 (0.1)	7 (0.1)	12 (0.2)	7 (0.1)

Percentage in parentheses. Level 1: no problems; 2: slight problems; 3: moderate problems; 4: severe problems; 5: extreme problems.

**Table 3:** Problem score distribution.

Problem Score	Possible States	2012	2013	2014	All
**5**	1	1213 (59.3)	1286 (63.4)	1240 (62)	3739 (61.6)
**6**	5	261 (12.8)	270 (13.3)	270 (13.5)	801 (13.2)
**7**	15	195 (9.5)	154 (7.6)	163 (8.1)	512 (8.4)
**8**	35	104 (5.1)	90 (4.4)	85 (4.2)	279 (4.6)
**9**	70	76 (3.7)	67 (3.3)	73 (3.6)	216 (3.6)
**10**	121	53 (2.6)	48 (2.4)	45 (2.2)	146 (2.4)
**11**	185	48 (2.3)	37 (1.8)	39 (1.9)	124 (2.0)
**12**	255	27 (1.3)	25 (1.2)	28 (1.4)	80 (1.3)
**13**	320	31 (1.5)	14 (0.7)	22 (1.1)	67 (1.1)
**14**	365	8 (0.4)	11 (0.5)	14 (0.7)	33 (0.5)
**15**	381	5 (0.2)	9 (0.4)	9 (0.4)	23 (0.4)
**16**	365	8 (0.4)	1 (0.0)	7 (0.3)	16 (0.3)
**17**	320	6 (0.3)	5 (0.2)	3 (0.1)	14 (0.2)
**18**	255	3 (0.1)	6 (0.3)	2 (0.1)	11 (0.2)
**19**	185	4 (0.2)	2 (0.1)	1 (0)	7 (0.1)
**20**	121	3 (0.1)	1 (0.0)	-	4 (0.1)
**21**	70	-	1 (0.0)	-	1 (0.0)
**22**	35	-	-	-	0 (0.0)
**23**	15	-	-	-	0 (0.0)
**24**	5	-	1 (0.0)	-	1 (0.0)
**25**	1	-	-	-	-
**All**	3125	2045	2028	2001	6074

Numbers as absolute values. Percentage in parentheses, if appropriate.

**Table 4:** Frequencies of most reported health states.

Health States	2012	2013	2014	All
11111 *	1213 (59.3)	1286 (63.4)	1240 (62.0)	3739 (61.6)
**11121**	178 (8.7)	167 (8.2)	160 (8.0)	505 (8.3)
**11112**	69 (3.4)	83 (4.1)	95 (4.7)	247 (4.1)
11122 *	75 (3.7)	43 (2.1)	53 (2.6)	171 (2.8)
**21121**	51 (2.5)	52 (2.6)	61 (3.0)	164 (2.7)
**21221**	29 (1.4)	21 (1.0)	28 (1.4)	78 (1.3)
**11113**	23 (1.1)	13 (0.6)	16 (0.8)	52 (0.9)
**11131**	26 (1.3)	15 (0.7)	13 (0.6)	54 (0.9)
**Others**	381 (18.6)	348 (17.2)	335 (16.7)	1064 (17.5)

Percentage in parentheses; * *p* < 0.05, chi-square test for statistically significant differences among all 3 years. Coding of health states: 1 = no problem; 2 = slight problems; 3 = some problems; 4 = severe problems; 5 = extreme problems. 1st digit: mobility; 2nd digit: self-care; 3rd digit: usual activities; 4th digit: pain/discomfort; 5th digit: anxiety/depression.

**Table 5:** Prevalence of diseases.

**Disease**	2012	2013	2014	All
**Musculoskeletal disease**	97 (4.7)	83 (4.1)	85 (4.2)	265 (4.4)
**Hypertension**	138 (6.7)	108 (5.3)	127 (6.3)	373 (6.1)
**Diabetes**	67 (3.3)	63 (3.1)	66 (3.3)	196 (3.2)
**Heart disease**	39 (1.9)	34 (1.7)	44 (2.2)	117 (1.9)
**Rheumatism**	28 (1.4)	32 (1.6)	32 (1.6)	92 (1.5)
**Four or more diseases**	0 (0)	2 (0.1)	3 (0.1)	5 (0.1)
**Three diseases**	6 (0.3)	4 (0.2)	4 (0.2)	14 (0.2)
**Two diseases**	50 (2.4)	46 (2.3)	58 (2.3)	154 (2.5)
**One disease**	251 (12.3)	208 (10.3)	214 (10.7)	673 (11.1)

Percentage in parentheses. No statistically significant differences found over the 3-year period (chi-square test, *p* < 0.05).

## Supplementary Materials

The following are available online at www.mdpi.com/1660-4601/13/3/343, Table S1: Representativity of data, Table S2: Age distribution, VAS values, aggregated samples 2012, 2013, and 2014.

## Abbreviations

The following abbreviations are used in this manuscript:

ANOVA: Analysis of variance
HRQoL: Health-related quality of life
VAS: Visual Analog Scale
