# Supplementary material for: EQ-5D-5L in the General German Population: Comparison and Evaluation of Three Yearly Cross-Section Surveys
Source: Int J Environ Res Public Health. 2016 Mar 21;13(3):343. doi: 10.3390/ijerph13030343 (PMC4809006; doi:10.3390/ijerph13030343)
Supplement: Supplementary file 1 [file ijerph-13-00343-s001.pdf]

Supplementary Materials: EQ-5D-5L in the General German Population: Comparison and Evaluation of Three Yearly Cross-Section Surveys

Manuel B. Huber, Peter Reitmeir, Martin Vogelmann and Reiner Leidl

Table S1. Representativity of data.

Age Group (Years)	Male Wort & Bild (%)	Male Census (%)	Difference	Female Wort & Bild (%)	Female Census (%)	Difference
15–19	6.6	6.1	0.5	5.1	5.5	−0.4
20–29	13.3	14.7	−1.4	14.4	13.4	1.0
30–39	16.4	14.2	2.1	16.5	13.2	3.3
40–49	18.3	19.9	−1.6	18.7	18.2	0.5
50–59	20.7	17.3	3.4	18.2	16.4	1.8
60–69	13.3	12.6	0.7	14.0	12.6	1.4
70–79	9.9	11.0	−1.1	10.2	12.7	−2.5
80–89	1.6	4.3	−2.7	2.9	8.0	−5.1

Census data available from [1].

Table S2. Age distribution, VAS values, aggregated samples 2012, 2013, and 2014.

Stratification	Gender	Age	N	Mean	SD	Confidence Interval Lower Bound	Confidence Interval Upper Bound
Overall	–	–	6074	84.3	15.9	83.9	84.7
Age group (years)	–	15–19	353	96.5	7.2	95.7	97.2
	–	20–29	842	93.5	9.1	92.9	94.1
	–	30–39	998	90.8	11.3	90.1	91.5
	–	40–49	1125	87.3	12.5	86.5	88.0
	–	50–59	1175	81.9	15.2	81.0	82.8
	–	60–69	829	76.2	15.8	75.1	77.2
	–	70–79	612	70.2	16.1	68.9	71.4
	–	80+	140	58.7	19.6	55.4	61.9
Gender	male	–	2859	85.7	15.3	85.1	86.3
	female	–	3215	83.1	16.3	82.5	83.7
Gender by age	male	15–19	188	97.0	6.2	96.2	97.9
	male	20–29	380	95.1	7.5	94.4	95.9
	male	30–39	468	92.3	10.1	91.4	93.2
	male	40–49	524	88.3	12.0	87.3	89.3
	male	50–59	591	83.4	15.0	82.2	84.6
	male	60–69	379	76.9	15.5	75.3	78.5
	male	70–79	283	70.9	15.5	69.1	72.7
	male	80+	46	57.5	15.7	53.0	62.0
	female	<20	165	95.9	8.2	94.6	97.1
	female	20–29	462	92.2	10.0	91.3	93.1
	female	30–39	530	89.5	12.1	88.5	90.6
	female	40–49	601	86.3	12.7	85.3	87.4
	female	50–59	584	80.4	15.4	79.1	81.6
	female	60–69	450	75.5	16.1	74.1	77.0
	female	70–79	329	69.6	16.6	67.8	71.3
	female	80+	94	59.3	21.3	55.0	63.6

References

1. Zensus-Statistisches Bundesamt. Zensus 2011. Available online: https://www.destatis.de/DE/Methoden/Zensus_/Zensus.html (accessed on 10 January 2016).

© 2016 by the authors; licensee MDPI, Basel, Switzerland. This article is an open access article distributed under the terms and conditions of the Creative Commons by Attribution (CC-BY) license (http://creativecommons.org/licenses/by/4.0/).
